# A Replanted Tooth

**Published:** 1867-10

**Authors:** W. M. Herriott

**Affiliations:** Zanesville, O.


### A REPLANTED TOOTH.

THE following case was reported by Dr. H. at the late meeting of the American Dental Association. It is peculiarly interesting, and the more so, as it is reported from careful record and observation. W.

EDITORS OF DENTAL REGISTER :—At the request of Dr. Watt, I put upon paper a short report of a case verbally reported by me at last meeting of the "American Dental Association."

Aug. 13th, 1857, Mrs. R——, aged eighteen years, came to my office to have the first right upper molar tooth removed. By taking hold of my hand and moving her head, she shifted the instrument so that instead of the molar I extracted the second bicuspid, a perfectly sound tooth. Then I extracted the aching tooth ; but the patient being so much annoyed by the loss of a perfect tooth, I concluded to replace it, which I did, after it had been out of the mouth about ten minutes. I first removed the ragged portions of the periosteum from the root ; then thoroughly cleansing the socket with pledgets of cotton and tepid water, I returned the tooth, closed the mouth and bandaged it to hold the tooth in place. I treated it in the usual way till the tooth was sufficiently firm in place to remove the bandage, which was in about three weeks. I saw the case occasionally for two weeks longer, when I dismissed the patient. I saw her every few months since, but heard no complaint, until about six months ago she came to me with the *same tooth* aching from *exposed nerve.*

*Zanesville, O.* W. M. HERRIOTT.

